# OncoSeg2D: A deep framework for semantic segmentation of lung cancer in 2D CT scans

**DOI:** 10.1371/journal.pone.0348719

**Published:** 2026-07-02

**Authors:** Luhang Yu, Xueshan Dong, Kai Fa, Yifan Li

**Affiliations:** 1 Senior Department of Oncology, Chinese PLA General Hospital, Beijing, China; 2 Department of Oncology, Beijing Chaoyang Integrative Medicine Rescue and First Aid Hospital, Beijing, China; 3 Department of Ultrasound Diagnosis, The Sixth Medical Center of Chinese PLA General Hospital, Beijing, China; 4 Department of Integrated Traditional Chinese and Western Medicine, Affiliated Hospital of Hebei University, Baoding, Hebei, China; Khalifa University of Science and Technology, UNITED ARAB EMIRATES

## Abstract

Lung cancer lesion segmentation in two-dimensional computed tomography (2D CT) images remains challenging due to blurred boundaries, heterogeneous morphologies, and annotation uncertainty, leading to unreliable delineations and reduced clinical usability. To address this research gap, we propose a novel 2D CT lung cancer semantic segmentation framework, OncoSeg2D, which explicitly tackles boundary ambiguity and morphological distortion through two complementary modules. Specifically, an Uncertainty-aware Boundary Modeling (UBM) module probabilistically represents tumor edges via learnable mean–variance estimation and gradient-weighted sampling, while a Morphology-Preserving Regularization (MPR) module constrains the segmentation with curvature, compactness, and convexity priors to maintain global shape consistency. The framework integrates these designs with multi-scale feature extraction from a SegFormer backbone and requires no additional annotations or three-dimensional (3D) reconstruction. Experiments conducted on the Medical Segmentation Decathlon Challenge dataset and the lung cancer segmentation dataset demonstrate that OncoSeg2D achieves IoU scores of 0.865 and 0.788, mIoU scores of 0.881 and 0.799, and Dice Similarity Coefficients (DSC) of 0.923 and 0.816, consistently outperforming conventional CNN-based models and mainstream Transformer-based methods. Compared with the SegFormer baseline, the proposed method improves mIoU by 3.8% and 4.0% on the two datasets, respectively, while reducing the Hausdorff distance from 5.61 to 3.41 and from 7.26 to 5.28, indicating superior boundary refinement and stronger global shape consistency. These results verify that explicitly integrating uncertainty modeling and morphological priors yields both higher accuracy and better interpretability. Overall, the proposed framework not only enhances segmentation accuracy but also improves clinical interpretability and reliability, offering a promising solution for lung cancer diagnosis assistance and therapeutic outcome monitoring.

## 1. Introduction

Lung cancer is one of the leading malignant tumors threatening human health, accounting for nearly 18% of all cancer-related deaths worldwide and remaining the foremost cause of cancer mortality according to the World Health Organization. The five-year survival rate remains below 20%, largely due to delayed detection and imprecise lesion delineation during clinical assessment [1]. Timely and accurate lesion segmentation is of great significance for computer-aided diagnosis (CAD), quantitative assessment of lesions, and treatment monitoring [2]. In modern clinical workflows, CAD systems assist radiologists by automating lesion identification, contouring, and volumetric quantification, thus reducing diagnostic workload and inter-observer variability while improving the reproducibility of therapeutic decisions. Accurate segmentation provides the foundation for radiomics feature extraction, tumor staging, and radiation therapy planning, directly influencing patient outcomes and follow-up evaluation [3]. 2D CT slices possess the advantages of large availability, broad coverage, and convenient acquisition, making it practically valuable to perform efficient automatic segmentation directly at the 2D level without relying on 3D reconstruction. Unlike regular organs or structured tissues, lung tumors in CT images often exhibit diverse morphologies, significant scale variations, low contrast, and blurred boundaries, which further intensify the challenges of robustness and interpretability in segmentation tasks. Despite long-standing clinical use, traditional semi-automatic or rule-based delineation strategies are often limited by hand-crafted features and strong assumptions about lesion appearance, making them brittle under heterogeneous scanners, varying reconstruction kernels, and highly irregular tumor shapes. These limitations frequently lead to unstable contours and poor generalization, particularly when lesions exhibit subtle contrast or complex spiculation patterns. Therefore, it is crucial to develop a learning-based segmentation paradigm that can adaptively capture complex imaging features while maintaining clinically meaningful contours [4].

In recent years, deep learning–based segmentation has become the dominant direction in medical image analysis because it can learn hierarchical representations directly from data, effectively modeling complex texture patterns and contextual cues that are difficult to encode with hand-crafted descriptors. In particular, convolutional architectures and Transformer-based encoders are capable of capturing local details and long-range dependencies, which is essential for delineating lesions with large appearance variability and ambiguous borders. However, directly applying existing deep models to lung tumor CT segmentation still faces notable gaps between algorithmic outputs and clinical-grade requirements, especially under noisy and heterogeneous imaging conditions. The first is boundary uncertainty: affected by imaging noise, low contrast, and inherent blurred transition zones of lesions, pixel-level hard-label supervision often induces prediction jitter and instability at the boundaries, forcing the network to make overconfident predictions in uncertain regions [5,6]. The second is morphological degradation: the purely pixel-wise classification objective lacks structural priors, leading to fragmented predictions, holes, disconnections, and non-convex shapes, which affect morphometric parameters and subsequent clinical interpretation. Moreover, despite the notable success of deep learning in lung cancer segmentation, challenges remain in achieving clinical-grade precision under heterogeneous imaging protocols, scanner variability, and lesion complexity. These limitations underscore the necessity of incorporating uncertainty estimation and morphology-aware priors into the segmentation framework to ensure both reliability and clinical interpretability [7]. Furthermore, although multi-scale context can alleviate local noise, in the absence of explicit modeling of uncertainty and global regulation of morphological consistency, predictions may still fluctuate at the edges and suffer from structural inconsistencies. Table 1 shows the impact of different backbone architectures on lung cancer segmentation.

**Table 1:** Comparison of different backbone networks for 2D CT lung cancer segmentation.

Backbone	Main Advantages	Main Disadvantages	Suitability
UNet	Simple structure, skip connections help preserve details, stable training.	Limited receptive field, hard to capture long-range dependencies; insufficient boundary modeling.	Partially suitable
ResNet50	Strong representation ability, residuals ease gradient vanishing.	Local convolution causes poor global context; sensitive to blurred boundaries.	Partially suitable
MiT-B2 (SegFormer)	Lightweight, efficient multi-scale extraction; balances small targets and global consistency.	Lacks explicit boundary modeling; needs extra regularization.	Suitable
ConvNeXt	Combines CNN + Transformer advantages; strong features, balanced efficiency/accuracy.	Transferability in medical imaging uncertain; weak boundary modeling.	Partially suitable
DenseNet121	Dense connections reuse features, rich fine-grained representation.	Many parameters, slower inference; weak global consistency.	Partially suitable

To bridge the above gaps, we design OncoSeg2D as a boundary- and morphology-aware 2D segmentation framework. Instead of introducing the method abruptly, we explicitly align the framework design with the two core challenges identified above: (i) boundary ambiguity requires uncertainty-aware modeling rather than deterministic hard-boundary fitting, and (ii) clinically credible delineation requires enforcing global morphological consistency beyond pixel-wise classification. To address these issues, the proposed OncoSeg2D consists of three components: a SegFormer MiT-B2 [8] backbone for extracting multi-scale semantic and boundary cues; Uncertainty-aware Boundary Modeling, which explicitly represents ambiguous boundaries with distributional parameterization (mean–variance) and sampling to characterize multiple feasible contours, while using gradient-field-based boundary-sensitive weighting to avoid overconfident fitting in uncertain transition zones; Morphology-preserving Regularization uses soft segmentation probabilities as carriers, introducing geometric–morphological operators such as level sets, curvature, area/perimeter compactness, convex hull consistency, and anisotropy of structure tensors. These operators perform projection-based updates in continuous geometric space, mapping the segmentation into a feasible set that satisfies priors such as connectivity, compactness, convexity, and preservation of spiculation, thereby achieving collaborative optimization of boundary refinement and global morphological consistency. This design requires no additional annotations and can output smoother and more reliable contours during inference.

The main contributions of this paper are summarized as follows:

(1) We propose the OncoSeg2D framework for 2D CT lung cancer segmentation, which simultaneously balances local boundary details and global structural consistency without relying on 3D reconstruction or additional supervision;
(2) We introduce uncertainty-aware boundary modeling, which elevates blurred boundaries from deterministic masks to probabilistic distributions, enabling the model to remain cautious in uncertain regions while being more confident in clear boundaries through mean–variance parameterization and gradient weighting;
(3) We present morphology-preserving regularization, which leverages level sets, curvature, compactness, convex hull consistency, and anisotropy as projection operators to achieve a progressive transformation from semantic representation to geometric regularization and finally to morphological refinement.

## 2. Related work

### 2.1 Medical image segmentation

Medical image segmentation has long been recognized as a critical foundation for computer-aided diagnosis and treatment planning, particularly in thoracic oncology where accurate delineation of lung nodules and lesions directly impacts diagnostic reliability and therapeutic outcomes. Traditional deep learning-based segmentation frameworks such as UNet and V-Net have achieved considerable success, yet their performance is often limited by boundary ambiguity, inter-patient variability, and complex morphological characteristics of pulmonary structures. Recent research has therefore focused on improving feature representation and spatial precision through network architectural innovations. For instance, Lu et al. [9] developed a deep learning-based lung nodule detection and recognition model that effectively combines multi-level contextual learning, while Wu et al. [10] proposed IAD-VNet to enhance feature aggregation and suppress false positives in pulmonary nodule segmentation. Similarly, Jiang et al. [11] introduced a boundary-aware dual-branch neural network that emphasizes contour-sensitive feature extraction to refine segmentation edges, and Lian et al. [12] integrated PET/CT data within a CFC-MSPCNN framework to exploit multimodal information for more accurate tumor localization.

In addition to architecture-level innovations, recent works have increasingly emphasized clinical relevance, interpretability, and robustness of segmentation models across varying imaging modalities and disease contexts. Xie et al. [13] demonstrated the clinical value of multiscale CT radiomics for differential diagnosis between benign and malignant lung nodules, highlighting the potential synergy between segmentation and radiomics analysis. Riahi et al. [14] further reviewed how machine learning and artificial intelligence are revolutionizing lung cancer management by enabling more personalized and precise treatment strategies. Beyond lung cancer, similar deep segmentation techniques have been extended to related thoracic diseases such as COVID-19, where Arora et al. [15] proposed a multi-label deep network capable of segmenting and detecting pulmonary abnormalities from chest radiographs. Collectively, these studies underscore a growing trend toward hybrid, context-aware, and clinically interpretable segmentation methods that bridge the gap between algorithmic accuracy and practical medical application.

### 2.2 Uncertainty modeling in medical image segmentation

Uncertainty modeling in medical image segmentation has gradually become a research hotspot, aiming to characterize the sources of uncertainty in model predictions under conditions such as boundary ambiguity, annotation variability, and limited training data. Kendall et al. [16] proposed to distinguish and model aleatoric and epistemic uncertainties within Bayesian deep learning, providing a foundational framework for tasks such as semantic segmentation. Subsequently, Kohl et al. [17] introduced the Probabilistic U-Net, which employs a conditional variational autoencoder to generate diverse segmentation outputs, thereby capturing images with multiple plausible solutions. Wang et al. [18] further combined test-time augmentation strategies to estimate uncertainty induced by observational noise in convolutional neural network-based segmentation. Mehrtash et al. [19] approached the problem from the perspective of model calibration, proposing confidence calibration methods to enhance the consistency between predicted probabilities and ground truth distributions, thus improving the reliability of uncertainty estimation. These methods collectively advanced uncertainty modeling from a theoretical concept to practical applications in medical image segmentation.

In recent years, extensive studies have focused on expanding the capacity of uncertainty modeling across diverse application scenarios. Zou et al. [20] provided a systematic review of uncertainty estimation methods in medical image segmentation, covering Bayesian networks, ensemble learning, probabilistic models, and test-time augmentation. Buddenkotte et al. [21] proposed a scalable ensemble-based framework for uncertainty quantification, which effectively improved reliability in large-scale segmentation tasks through model calibration. Roshanzamir et al. [22] linked inter-rater variability with both aleatoric and epistemic uncertainties, revealing how subjective annotation differences influence the trustworthiness of segmentation results. Huang et al. [23] integrated Bayesian U-Net with active learning strategies to guide sample selection in multi-label dental CT segmentation tasks based on uncertainty. Meanwhile, Li et al. [24] introduced the QUBIQ challenge, which provides benchmark datasets for uncertainty quantification, and Chlap et al. [25] conducted empirical studies using a 3D probabilistic U-Net on small-scale radiotherapy trial datasets. Collectively, these works demonstrate that uncertainty modeling not only enhances segmentation performance and generalization but also provides critical insights into the trustworthiness and interpretability of model predictions in clinical scenarios.

### 2.3 Morphology-aware regularization and structural priors

In medical image segmentation tasks, incorporating morphological priors and structural constraints has been regarded as an effective approach to enhance the plausibility of predictions and maintain structural consistency. Zheng et al. [26] modeled conditional random fields as recurrent neural networks and proposed CRF-RNN, which can be trained end-to-end to refine segmentation boundaries and ensure global consistency. Krähenbühl et al. [27] developed an efficient inference method for fully connected CRFs, which preserves fine boundary details through high-dimensional filtering. Oktay et al. [28] proposed Anatomically Constrained Neural Networks (ACNNs), embedding prior anatomical shapes into segmentation models to guarantee anatomical consistency. Kervadec et al. [29] designed a boundary-based loss function to alleviate contour deficiencies in highly imbalanced segmentation tasks. Karimi et al. [30] optimized the Hausdorff distance, explicitly reducing the maximum deviation between predictions and ground truth boundaries, thereby improving the geometric stability of segmentation results.

Further studies have introduced topological constraints and geometric regularization into deep learning frameworks to strengthen structural consistency. Hu et al. [31] proposed a topology-preserving segmentation method, where topological constraints were integrated into the training process to prevent connectivity violations. Clough et al. [32] introduced a topological loss function based on persistent homology to ensure that segmentation predictions align with global topological priors. Weigert et al. [33] proposed a star-convex polyhedra-based shape modeling method for microscopy image segmentation, enforcing geometric constraints to guarantee morphological plausibility. Chen et al. [34] integrated Euler’s elastica model with deep networks, achieving learnable morphological regularization based on curvature and length. Kang et al. [35] provided a comprehensive survey on fast algorithms for elastica-based models, offering systematic insights into elastica priors for image segmentation. These studies collectively demonstrate that morphological priors and structural constraints not only improve the robustness and interpretability of segmentation results but also provide crucial solutions to address complex boundaries and morphologically implausible predictions.

### 2.4 Summary and motivation

In summary, while existing research has made substantial progress in generic medical image segmentation, studies specifically targeting lung nodule and lesion segmentation in CT images still face persistent challenges. Conventional CNN-based methods often struggle with blurred and irregular tumor boundaries caused by low contrast and imaging noise, leading to over-smoothed or discontinuous contours that compromise diagnostic accuracy. Transformer-based approaches, though capable of capturing long-range dependencies, tend to neglect local structural priors and thus suffer from morphological distortion and fragmented predictions in complex thoracic regions. Moreover, most uncertainty-aware or morphology-regularized methods are designed for general anatomical structures rather than the highly heterogeneous and irregular patterns of lung tumors, resulting in limited generalization to real-world clinical cases. To bridge this gap, the proposed OncoSeg2D framework integrates uncertainty-aware boundary modeling and morphology-preserving regularization within a SegFormer backbone, effectively coupling probabilistic boundary refinement with global shape constraint. This design enables the model to achieve precise boundary delineation and structural coherence simultaneously, thereby enhancing the reliability, interpretability, and clinical applicability of 2D CT lung cancer segmentation.

## 3. Method

### 3.1 Overall model architecture

**Fig 1:**
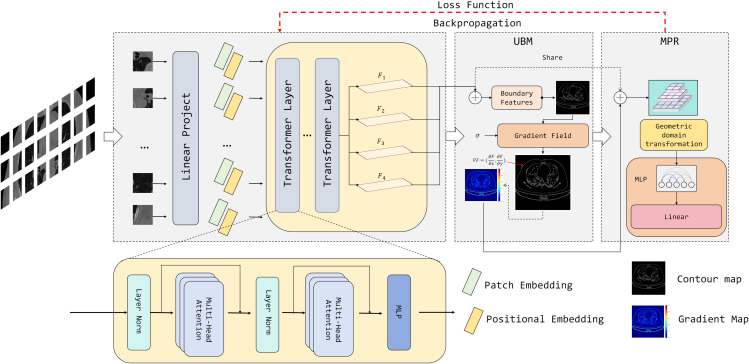
The overall framework of OncoSeg2D employs SegFormer MiT-B2 as the backbone to extract multi-scale features, incorporates UBM for uncertainty-aware boundary modeling, and integrates MPR for morphology-preserving regularization. This design enhances both boundary refinement and global structural consistency in lung cancer CT segmentation.

The overall structure of the OncoSeg2D framework proposed in this paper is shown in the Fig 1. Its core consists of three components: a feature extraction backbone network (Backbone), uncertainty-aware boundary modeling, and morphology-preserving regularization. The Backbone uses SegFormer’s MiT-B2 as the backbone network to extract semantic and boundary features from lung cancer CT slices at multiple scales, while the key novelty of OncoSeg2D lies beyond the backbone architecture. Specifically, SegFormer-style Transformer models primarily improve segmentation by designing efficient multi-scale feature encoding and lightweight decoding, but they typically treat boundaries as deterministic targets and do not explicitly quantify boundary ambiguity in medical lesions. In contrast, OncoSeg2D introduces an uncertainty-aware boundary modeling module that represents boundary predictions as a probabilistic distribution and explicitly captures boundary ambiguity, enabling more reliable boundary learning under weak contrast and irregular lesion contours. Moreover, although edge-aware Transformer variants such as EDAFormer enhance boundary perception through explicit edge/attention designs, they still focus on strengthening edge responses in a deterministic manner and lack an explicit mechanism to model boundary uncertainty and to constrain the predicted masks to satisfy clinically plausible morphology. OncoSeg2D further incorporates morphology-preserving regularization to enforce shape-consistent predictions, which complements uncertainty-aware boundary modeling by reducing unrealistic fragmentations and distortions, thereby forming a boundary-uncertainty–morphology synergy that is not present in SegFormer or EDAFormer.

Given an input 2D CT image $I\in {\mathbb{R}}^{H\times W}$, it first passes through a linear projection layer (Patch Embedding) to divide the image into fixed-size patches and project them into a low-dimensional feature space:


$$\begin{array}{c} X_{0}=PatchEmbed(I),\;X_{0}\in {\mathbb{R}}^{\frac{H}{P}\times \frac{W}{P}\times C}, \end{array}$$ (1)

where *P* is the patch size and *C* is the embedding dimension.

Next, MiT-B2 progressively extracts multi-scale features through four stages (Stages 1–4) of Transformer encoders, each consisting of an attention layer and a feed-forward network (FFN). In the *l*th layer, the input feature $X_{l-1}$ is updated via the multi-head attention (MHA) mechanism and residual connections to:


$$\begin{array}{c} X_{l}^{\prime }=X_{l-1}+MHA(X_{l-1}), \end{array}$$ (2)

Then it is further transformed by the feedforward network:


$$\begin{array}{c} X_{l}=X_{l}^{\prime }+FFN(X_{l}^{\prime }). \end{array}$$ (3)

After four stages, a set of feature maps at different scales is obtained:


$$\begin{array}{c} \{F_{1},F_{2},F_{3},F_{4}\}=MiT\text{-}B2(X_{0}), \end{array}$$ (4)

Where $F_{i}$ These correspond to low-level, fine-grained boundary features and high-level semantic features, respectively, providing a representational foundation for subsequent uncertainty-aware boundary modeling and morphology-preserving regularization.

In this architecture, the Backbone module is responsible for capturing the global context and local boundary patterns of CT slices. These multi-scale features not only enhance sensitivity to fuzzy tumor boundaries but also provide prior support for morphological constraints. Subsequently, the uncertainty-aware boundary modeling performs probabilistic boundary modeling in fuzzy regions, while morphology-preserving regularization constrains the morphological plausibility of the segmentation results through regularization, thereby achieving refined and structurally consistent lung cancer segmentation.

### 3.2 Uncertainty-aware boundary modeling

**Fig 2:**
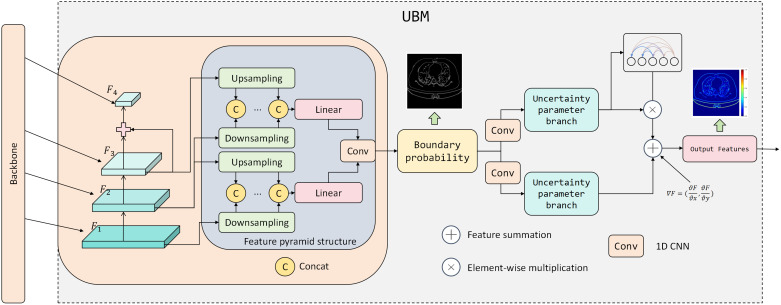
Schematic structure of the UBM module. Multi-scale features from the backbone are fused through a pyramid structure to generate the initial boundary probability representation, which is then combined with uncertainty parameter branches and gradient field information to produce feature representations that incorporate both boundary sensitivity and uncertainty modeling.

In lung cancer CT segmentation, tumor boundaries often exhibit inherent ambiguity, making it difficult even for experienced physicians to accurately delineate pixel-level contours. This ambiguity arises not only from imaging device noise and contrast deficiencies, but also from the morphological uncertainty of the lesions themselves. If traditional one-hot labeling is used for supervision, the model will forcibly learn hard labels at the boundaries, resulting in jittery predictions. Therefore, in OncoSeg2D, we propose Uncertainty-Aware Boundary Modeling to explicitly model this ambiguity in feature space, making the model’s representation of boundary transitions more flexible and robust. Its module architecture is shown in Fig 2.

The idea behind uncertainty-aware boundary modeling is to leverage the multi-scale features $\left\{F_{1}F_{2}F_{3}F_{4}\right\}$ output by the backbone to generate a probability distribution for the boundaries rather than a single binary prediction. First, we align and fuse the feature maps at a unified scale to obtain the fused features:


$$\begin{array}{c} F=\Phi (F_{1}F_{2}F_{3}F_{4}) \end{array}$$ (5)

Where Φ(·) represents the multi-scale feature fusion operation, which ensures that the model can simultaneously utilize low-level, fine-grained boundary information and high-level semantic context information.

After obtaining the fused features, we want to map them to a boundary response space. To this end, we introduce the boundary saliency function:


$$\begin{array}{c} B=\sigma (W_{b}*F) \end{array}$$ (6)

Where $W_{b}$ is the convolution kernel, * represents the convolution operation, and σ(·) is the sigmoid function, which constrains the output to the range [0,1] and represents the boundary probability.

However, boundary probabilities alone are insufficient to describe uncertainty. Therefore, we further introduce mean and variance parameterized features through variational methods:


$$\begin{array}{c} \mu =W_{\mu }*F \end{array}$$ (7)

$$\begin{array}{c} \sigma^{2}=ReLU(W_{\sigma }*F) \end{array}$$ (8)

Where μ and $\sigma^{2}$ represent the mean and variance of the boundary region, respectively, reflecting the degree of uncertainty in the boundary location.

Based on the above parameterization, we can sample the potential boundary distribution in the feature space:


$$\begin{array}{c} Z=\mu +\sigma ⊙\epsilon \end{array}$$ (9)

Where ϵ~𝒩(01) is Gaussian noise, and ⊙ represents element-wise multiplication. This sampling mechanism enables the model to generate different possible contours at the boundary, thereby simulating the differences in manual annotations.

Furthermore, to further refine boundary modeling, we combine the original boundary probability with the sampled latent representation:


$$\begin{array}{c} \tilde{B}=\alpha B+(1-\alpha )\sigma (Z) \end{array}$$ (10)

Where α is the balancing coefficient, and σ(·) serves as the normalization function. This equation indicates that the final boundary estimate is a weighted combination of the deterministic boundary probability and the uncertain sampling results.

At the spatial level, boundary uncertainty can be expressed using a gradient field. We define the gradient magnitude of the fused feature as:


$$\begin{array}{c} G=\sqrt{{\left(\frac{\partial F}{\partial x}\right)}^{2}+{\left(\frac{\partial F}{\partial y}\right)}^{2}} \end{array}$$ (11)

Where (*x y*) is the spatial coordinate. This gradient field characterizes the region with the most dramatic boundary changes, which often corresponds to the transition zone at the edge of the tumor.

Combining the uncertainty parameter σ with the gradient field, we can further construct a boundary-sensitive weight:


$$\begin{array}{c} W_{u}=\exp(-\sigma^{2})⊙G \end{array}$$ (12)

This weight indicates that the model is more confident about the boundary location in regions with low uncertainty and large gradients, while remaining flexible in regions with high uncertainty.

To explicitly separate boundary and non-boundary regions in the feature space, we introduce a boundary mask:


$$\begin{array}{c} M=𝕀(\tilde{B}>\tau ) \end{array}$$ (13)

Where τ is the threshold and 𝕀(·) is the indicator function. This mask divides the feature map into potential boundary and non-boundary regions.

Furthermore, we weight the fused features *F* under the mask constraint:


$$\begin{array}{c} F_{b}=M⊙F\;F_{nb}=(1-M)⊙F \end{array}$$ (14)

Where $F_{b}$ represents boundary features, and $F_{nb}$ represents non-boundary features. By explicitly separating them, the model can learn stronger discriminative capabilities for boundary regions during training.

Finally, to maintain boundary consistency across scales, we align the boundary representations of features at each scale:


$$\begin{array}{c} \hat{B}=\Psi ({\tilde{B}}_{1}{\tilde{B}}_{2}{\tilde{B}}_{3}{\tilde{B}}_{4}) \end{array}$$ (15)

Where Ψ(·) represents the multi-scale fusion function, and ${\tilde{B}}_{i}$ represents the uncertainty boundary estimate obtained at the *i*th scale. This operation ensures consistent boundary prediction at different resolutions.

In summary, the uncertainty-aware boundary modeling transforms boundary ambiguity in lung cancer CT images into explicit uncertainty modeling through probabilistic boundary modeling, mean-variance parameterization, random sampling, and gradient guidance, thereby improving the model’s robustness and generalization under complex boundary conditions. Tightly integrated with the multi-scale features extracted by Backbone, the uncertainty-aware boundary modeling not only avoids overfitting to hard labels but also produces smoother and more clinically meaningful segmentation results during inference.

### 3.3 Morphology-preserving regularization

**Fig 3:**
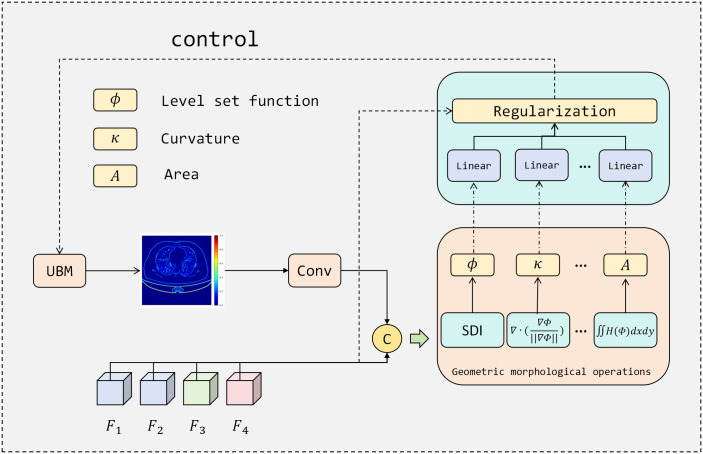
Schematic diagram of the MPR module architecture. It first utilizes uncertainty-aware boundary modeling output and multi-scale features to generate geometric representations such as level set functions, curvature, and area. Subsequently, through geometric morphological operations and regularization units, structural priors are embedded in the segmentation process to maintain morphological plausibility.

In 2D CT slices, the visual morphology of lung cancer tumors often exhibits a coexistence of an overall nearly elliptical shape and localized spurs, with breaks and gaps often present at their boundaries. Relying solely on pixel-by-pixel classification often results in illogical fragmentation and morphological distortion. Therefore, in OncoSeg2D, we introduce a morphology-preserving regularization module without changing the basic decoder architecture. This module uses geometric and morphological operators to enforce consistent constraints on multi-scale semantic features from Backbone and boundary cues from the uncertainty-aware boundary modeling. Using soft segmentation probabilities as a carrier and representations such as level sets and structure tensors, it performs projective updates in the feature and mask spaces. This approach, without explicitly introducing supervision, establishes soft constraints on connectivity, density, convexity, and spur preservation. Its module architecture is shown in Fig 3.

First, the fused features *F* and the uncertainty boundary estimate $\tilde{B}$ from the uncertainty-aware boundary modeling are combined to form the initial region probability *P*. This step serves only as the input representation for subsequent geometric operations:


$$\begin{array}{c} P=\sigma \;\left(W_{p}*\left(F⊕\tilde{B}\right)\right) \end{array}$$ (16)

Where ⊕ represents feature-level concatenation, * represents convolution, and σ is the compression function. The resulting *P* encodes both global semantics and boundary confidence.

To perform geometric morphological computation, we use the signed distance transform to map *P* to the level set function ϕ, where positive values represent the inside of the target and negative values represent the outside:


$$\begin{array}{c} \phi =SDT\;\left(P\right) \end{array}$$ (17)

ϕ provides a continuously differentiable isosurface representation, facilitating a unified measure of curvature, connectivity, and contour length.

The curvature of ϕ can be used to characterize the curvature of local boundaries, thereby selectively smoothing noise-induced aliasing:


$$\begin{array}{c} \kappa =\nabla \cdot \left(\frac{\nabla \phi }{\left\|\nabla \phi \right\|}\right) \end{array}$$ (18)

κ is a curvature scalar field whose magnitude indicates the strength of local convexity and concavity.

Combined with the boundary uncertainty weight $W_{u}$ provided by the uncertainty-aware boundary modeling, we use a first-order explicit update to perform a boundary-sensitive regularized flow on ϕ to suppress unreasonable small fluctuations and stop at the clear boundary:


$$\begin{array}{c} \phi_{t+1}=\phi_{t}+\eta \;W_{u}\;\kappa \end{array}$$ (19)

Where η is the step size, $W_{u}$ comes from the uncertainty-aware boundary modeling gradient uncertainty map, and reduces the flow rate in high uncertainty regions to avoid oversmoothing truly fuzzy boundaries.

At the global scale, we define compactness metrics using area and contour length. The higher the compactness, the closer the contour is to a reasonably dense form:


$$\begin{array}{c} A=\iint H\;\left(\phi \right)\;dx\;dy \end{array}$$ (20)

*A* is the target area calculated by the Heaviside function *H*.


$$\begin{array}{c} L=\iint \delta \;\left(\phi \right)\left\|\nabla \phi \right\|\;dx\;dy \end{array}$$ (21)

*L* is the contour length calculated by the Dirac function δ.


$$\begin{array}{c} 𝒞=4\pi AL^{-2} \end{array}$$ (22)

𝒞 is a classic compactness measure, used to indicate whether the target consists of long, fragmented edges.

To describe the gap-filling tendency, we introduce a convexity measure based on the convex hull. First, we calculate the convex hull area of the probability map:


$$\begin{array}{c} A_{h}=Area\;\left(Hull\;\left(P\right)\right) \end{array}$$ (23)

$A_{h}$ is the minimum convex hull cover area.


$$\begin{array}{c} 𝒱=A\;A_{h}^{-1} \end{array}$$ (24)

𝒱 represents the degree of consistency between the area cover and the convex hull, and can be used to guide the closing of small gaps at credible boundary locations.

At the local structural level, we measure directional anisotropy using the structure tensor to characterize radial morphologies such as burrs and thin protrusions:


$$\begin{array}{c} J=K_{\rho }*\left(\nabla P\;\nabla P^{⊤}\right) \end{array}$$ (25)

Where $K_{\rho }$ is a Gaussian kernel of scale ρ, and the eigendecomposition of *J* gives the principal directional energy.


$$\begin{array}{c} r=\lambda_{1}\;\lambda_{2}^{-1} \end{array}$$ (26)

*r* is the anisotropy ratio, and $\lambda_{1}\geq \lambda_{2}$ are the eigenvalues of *J*. When *r* is large, it indicates the presence of thin radial structures, and the smoothing strength needs to be reduced to preserve the semantics of the burrs.

By integrating multiple morphological variables such as curvature, compactness, convexity, and anisotropy, we project the initial probability *P* onto a feasible set that satisfies the morphological prior using a projection scheme, resulting in a morphologically consistent soft mask $\hat{P}$:


$$\begin{array}{c} \hat{P}={𝒫}_{morph}\;\left(P|\phi \;\kappa \;𝒞\;𝒱\;r\right) \end{array}$$ (27)

Where ${𝒫}_{morph}$ represents the morphological projection operator, which internally performs curvature-guided smoothing on the isosurface in a small number of iterations, performs a weak closure along the convex hull’s trusted directions, and adaptively adjusts the balance between smoothing and detail preservation based on the spatial distribution of *r*.

At the implementation level, the above process follows a unidirectional information flow from semantics to geometry and back to probability. First, the multi-scale features generated by Backbone and the boundary confidences of the uncertainty-aware boundary modeling form a probabilistic representation of the region. This is then transformed into a continuous geometric domain via a distance transform. Within this domain, local smoothness is described using curvature, global density using area, length, and compactness, gap-filling propensity using convexity, and radial and burr directionality using a structure tensor. Finally, projection is performed back to the probabilistic domain to obtain $\hat{P}$. This design allows semantics and morphology to interact on a unified intermediate representation, where the uncertainty-aware boundary modeling weight field $W_{u}$ controls the regularization flow, maintaining tolerance near true blur boundaries while suppressing noise artifacts.

From another perspective, morphology-preserving regularization and uncertainty-aware boundary modeling complement each other: the uncertainty-aware boundary modeling provides probabilistic uncertainty modeling of boundaries at the pixel-to-probabilistic level, while the morphology-preserving regularization performs global shape uniformity and restoration at the geometric-to-regional level. By collaboratively utilizing ϕ,κ,𝒞,𝒱,andr, we introduce structural properties that balance connectivity, density, convexity, and directionality to the segmentation results without changing the backbone architecture. The resulting $\hat{P}$ is both semantically informed and consistent with morphological priors, making it easy to perform subsequent thresholding or integrate with other modules.

### 3.4 Algorithm novelty and differences from other Transformer architecture segmentation algorithms

The proposed OncoSeg2D framework introduces a structurally novel design that diverges from existing Transformer-based segmentation models in three key aspects. First, instead of solely relying on self-attention for spatial feature refinement as in classical architectures such as TransUNet or Swin-Unet, OncoSeg2D explicitly integrates an uncertainty-aware boundary modeling mechanism that probabilistically characterizes edge ambiguity through learnable mean–variance estimation, thereby improving delineation of irregular tumor margins. Second, it incorporates a morphology-preserving regularization module that imposes geometric constraints derived from curvature, compactness, and convexity priors, ensuring global shape consistency that standard Transformer decoders fail to guarantee. Finally, the framework achieves a synergistic balance between pixel-level accuracy and topology-level robustness via joint optimization of uncertainty-aware boundary modeling and morphology-preserving regularization branches, enabling stable training and enhanced generalization across heterogeneous CT datasets. Collectively, these innovations differentiate OncoSeg2D from prior Transformer-based segmentation networks by embedding explicit structural reasoning and shape-aware constraints into the Transformer learning paradigm.

### 3.5 Dataset

The experimental datasets were collected from two publicly accessible sources. The first dataset was obtained from the Medical Segmentation Decathlon (MSD) lung task, which provides paired 3D CT volumes and expert annotations for lung lesions. We included patient cases that (i) contained complete CT volumes with corresponding voxel-wise lesion masks, and (ii) were successfully parsed without missing slices or corrupted metadata; cases with incomplete annotations or severe artifacts were excluded. The MSD lung task contains 96 cases in total, including 64 training cases and 32 official test cases; however, the 32 test cases are not publicly released with annotations, and thus we used only the 64 publicly available training cases in our experiments. Among these 64 training cases, 1 case (1.56%) was excluded due to a parsing failure, resulting in 63 eligible patient cases. All 63 eligible cases were used to avoid selection bias.

Each 3D CT volume was resampled to an isotropic spacing of 1.0 mm (linear interpolation for images and nearest-neighbor interpolation for masks), followed by HU clipping to [−1000, 400] to suppress air and high-density structures. The volumes were then sliced along the axial plane into 2D images. To ensure that 2D training focuses on clinically relevant regions while preserving local anatomical continuity, we retained slices containing visible lesions and additionally included a symmetric context window of K adjacent slices above and below each lesion slice. This strategy reduced the dominance of non-lesion slices and mitigated extreme class imbalance. In total, 8,640 2D slices were generated. Each slice was normalized before being fed into the 2D network. Slice-wise normalization was adopted to reduce inter-scan intensity shifts caused by heterogeneous acquisition protocols, which is common in CT-based 2D segmentation. To prevent data leakage, the dataset was split strictly at the patient level into training/validation/testing sets with a ratio of 7:1:2, ensuring that slices from the same patient appeared in only one split. Random flipping, rotation, and contrast perturbation were applied for augmentation.

The second dataset was collected from a Kaggle lung cancer segmentation dataset, which originally contained 2,535 2D CT images with corresponding expert-annotated lesion masks. Unlike MSD, this dataset is released directly as 2D samples rather than full 3D volumes; therefore, the total number of slices is lower even though the dataset aggregates images from multiple public sources. To reduce potential overlap between the two datasets and avoid evaluation bias, we further excluded those 2D slices that were visually identified as originating from MSD. This filtering process was performed manually, by inspecting and removing the relevant slices one by one. After removing these MSD-related samples, 1,933 2D CT images were retained in the final second dataset. Following the same approximate 7:1:2 split ratio, 1,353 images were used for training, 193 for validation, and 387 for testing. All images underwent the same intensity normalization and geometric/photometric augmentations as those used for MSD to maintain a consistent preprocessing pipeline.

**Fig 4:**
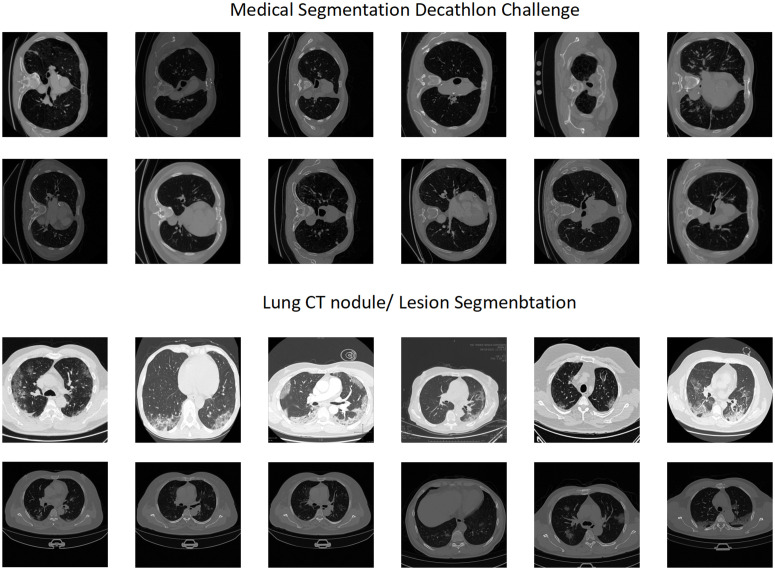
Medical Segmentation Decathlon Challenge & lung cancer segmentation dataset examples.

Furthermore, we randomly selected representative examples from both datasets for visualization in Fig 4. This figure is provided solely to illustrate the overall data appearance and typical imaging characteristics of the datasets used in this study, so that readers—especially those unfamiliar with these datasets—can quickly understand the visual style and acquisition variability and more easily assess whether these two datasets are suitable for their own subsequent research or application scenarios.

### 3.6 Experimental setup

In this study, we evaluated the OncoSeg2D framework using a publicly available lung cancer CT segmentation dataset. All experiments were conducted on the same hardware environment and maintained a consistent training configuration to ensure fairness. Training and inference were implemented in PyTorch, using the SegFormer MiT-B2 backbone network [8]. To ensure the reliability of the reported results, we adopted a five-fold cross-validation protocol, and the final performance was reported as the mean and standard deviation across the five folds. All hyperparameter/model selection was performed within the training and validation partitions of each fold only, and the corresponding held-out fold was used solely for final evaluation. We did not conduct an automated hyperparameter search; instead, we followed a controlled manual tuning protocol over a small set of candidate configurations. Due to the computational training cost, we did not perform large-scale or wide-range hyperparameter tuning, and focused on a limited but representative set of settings. All tuning runs followed the same five-fold cross-validation setting, and we trained models on the training partition while selecting configurations based only on validation performance within each fold. During tuning, the model architecture, preprocessing, and augmentation were kept unchanged, and we varied one factor at a time while keeping all other settings fixed. The final training configuration was chosen according to the best validation segmentation performance (primary criterion: IoU). Although OncoSeg2D exhibits slightly higher architectural complexity compared to certain lightweight frameworks such as SegMan, its integration of uncertainty-aware boundary modeling and morphology-preserving regularization provides distinct advantages in clinical scenarios that demand high boundary fidelity and structural interpretability. Specifically, the model demonstrates superior robustness when dealing with irregular, low-contrast lesions and offers more reliable shape-consistent predictions, which are essential for quantitative assessment and treatment planning. The specific model hyperparameters are shown in Table 2.

**Table 2:** Experimental model hyperparameter settings.

Training hyperparameters	Parameters
Backbone	SegFormer MiT-B2
Input size	512×512
Batch size	16
Optimizer	AdamW
Initial learning rate	$1\times 10^{-4}$
Learning Rate Scheduler	Cosine Annealing
Weight Decay	$1\times 10^{-2}$
Number of Training Episodes	200
Data Augmentation	Random Rotation, Flipping, and Intensity Normalization

### 3.7 Evaluation metric

In medical image segmentation, common evaluation metrics include Intersection over Union (IoU), mean IoU (mIoU), Hausdorff Distance (HD), Dice Similarity Coefficient (DSC), and mean Accuracy (mAcc). These metrics jointly assess the overlap, boundary accuracy, and classification consistency between predicted and ground-truth regions.

IoU quantifies the overlap between prediction *P* and ground truth *G*:


$$\begin{array}{c} IoU=\frac{\left|P\cap G\right|}{\left|P\cup G\right|} \end{array}$$ (28)

mIoU represents the mean of foreground and background IoUs:


$$\begin{array}{c} mIoU=\frac{1}{2}(IoU_{fg}+IoU_{bg}) \end{array}$$ (29)

HD measures the maximum boundary deviation:


$$\begin{array}{c} HD(P,G)=\max\left\{\underset{p\in \partial P}{\sup}\underset{g\in \partial G}{\inf}d(p,g),\underset{g\in \partial G}{\sup}\underset{p\in \partial P}{\inf}d(g,p)\right\}. \end{array}$$ (30)

In practice, we report the Hausdorff distance computed using the 95^th^ percentile of all point-to-boundary distances to reduce sensitivity to outliers.

DSC, equivalent to the F1-score, evaluates region overlap:


$$\begin{array}{c} DSC=\frac{2|P\cap G|}{\left|P|+|G\right|} \end{array}$$ (31)

mAcc denotes average pixel-level accuracy:


$$\begin{array}{c} mAcc=\frac{1}{2}\left(\frac{TP}{TP+FN}+\frac{TN}{TN+FP}\right) \end{array}$$ (32)

In summary, IoU and DSC focus on overlap quality, HD on boundary precision, and mIoU and mAcc on overall classification balance, providing a comprehensive assessment of segmentation performance.

## 4. Experimental results and analysis

### 4.1 Experimental results of evaluation indicators changing with epoch

**Fig 5:**
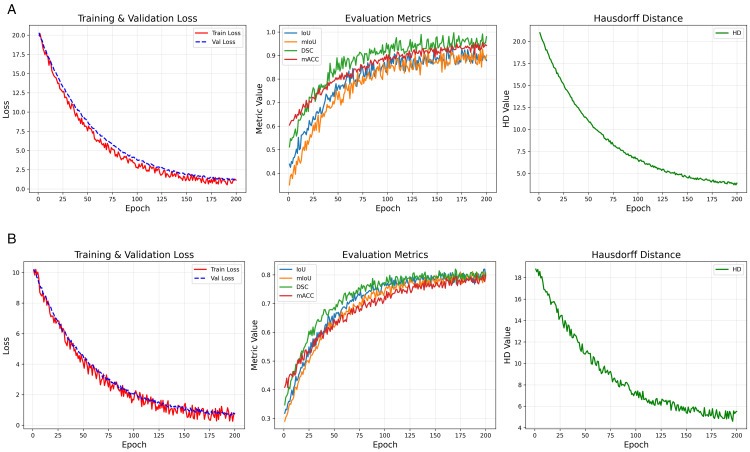
Evaluation indicators and loss curves on two datasets. **(a)** Evaluation indicators and loss curves on the Medical Segmentation Decathlon dataset. In this study, the Hausdorff Distance was computed on the validation set using the 95th percentile Hausdorff Distance metric. **(b)** Evaluation indicators and loss curves on the lung cancer segmentation dataset. In this study, the Hausdorff Distance was computed on the validation set using the 95th percentile Hausdorff Distance metric.

First, this paper presents the evaluation metrics and loss changes across epochs during training on the two datasets, as shown in Fig 5. The training process was conducted for the full scheduled number of epochs without employing an early stopping strategy. The final model was selected by choosing the epoch that achieved the highest validation IoU after convergence.

Experimental results show that both training and validation losses for both datasets show a rapid decline with increasing iterations, ultimately reaching a plateau, demonstrating good model convergence during training. Corresponding evaluation metrics (such as IoU, DSC, and mAcc) continue to rise and remain at high levels, demonstrating the effective improvement in the model’s segmentation performance. Furthermore, the Hausdorff distance gradually decreases, further verifying the model’s refined improvements and stability in boundary localization.

### 4.2 Comparison of experimental results with other models

To comprehensively evaluate the effectiveness of our proposed method, we compared OncoSeg2D with several representative segmentation models using the same dataset and training configuration. These included a classic convolutional neural network architecture (UNet++ [36]), a multi-scale feature aggregation model (DeepLabv3+ [37], nnU-net [38]), Transformer-based methods (Mask2Former [39], SegFormer [8], SegNeXt [40], VWFormer [41], and EDAFormer [42]), and a recently proposed hybrid architecture (SegMan [43]). These comparisons covered a diverse range of designs, from traditional CNNs to novel Transformers and hybrid architectures. This helped validate the competitiveness and applicability of our method across various mainstream frameworks.

We first present experimental results on the Medical Segmentation Decathlon Challenge dataset, as shown in Table 3.

**Table 3:** Experimental results on the Medical Segmentation Decathlon Challenge dataset (mean ± standard deviation across five-fold cross-validation).

Method	IoU	mIoU	HD ↓	DSC	mAcc	Params (M)	Latency (ms)
UNet++ [36]	0.768±0.032	0.779±0.030	7.34±0.71	0.829±0.029	0.809±0.034	9.2	12.5
DeepLabv3+ [37]	0.792±0.028	0.804±0.026	6.57±0.63	0.849±0.027	0.826±0.030	43.5	12.7
nnU-Net [38]	0.844±0.021	0.857±0.019	4.91±0.42	0.892±0.019	0.872±0.021	31.2	18.6
Mask2Former [39]	0.818±0.039	0.832±0.036	6.03±0.88	0.871±0.034	0.844±0.041	61.0	25.3
SegFormer [8]	0.830±0.034	0.843±0.031	5.61±0.74	0.881±0.030	0.855±0.034	64.0	14.3
SegNeXt [40]	0.839±0.027	0.853±0.024	5.24±0.55	0.888±0.024	0.864±0.027	48.9	16.1
VWFormer [41]	0.847±0.031	0.861±0.028	4.71±0.63	0.896±0.027	0.877±0.031	68.9	20.8
EDAFormer [42]	0.854±0.024	0.869±0.022	4.29±0.47	0.904±0.022	0.885±0.025	65.8	19.4
SegMan [43]	0.858±0.029	0.873±0.026	4.05±0.61	0.910±0.025	0.891±0.029	92.6	30.9
Ours	0.865±0.017	0.881±0.015	3.41±0.31	0.923±0.015	0.901±0.017	67.2	17.4

As shown in Table 3, the proposed OncoSeg2D outperforms existing mainstream models across all evaluation metrics. Compared to classic CNN architectures (such as UNet++ and DeepLabv3+), this method significantly improves IoU, mIoU, and DSC, while significantly reducing HD. This demonstrates that OncoSeg2D is able to better model boundary locations while maintaining overall segmentation accuracy. This suggests that relying solely on convolutional architectures fails to fully capture the complex boundary characteristics of lung lesions. The proposed uncertainty-aware boundary modeling plays a key role in addressing boundary ambiguity and annotation uncertainty.

Further comparison with Transformer-based methods (Mask2Former, SegFormer, SegNeXt, VWFormer, EDAFormer, and SegMan) reveals that, in addition to its global modeling capabilities, OncoSeg2D significantly improves the structural consistency and morphological plausibility of its segmentation results by introducing morphology-preserving regularization. MPR’s contributions to geometric constraints and topology preservation enable the model to achieve superior numerical performance while also effectively avoiding undesirable morphologies such as fragmentation or gaps in the generated results. Consequently, this method demonstrates enhanced robustness and clinical application potential in addressing the two core challenges of blurred boundaries and complex morphology in lung cancer CT images.

The experimental results of lung cancer segmentation Segmentation dataset are further given, and the experimental results are shown in Table 4.

**Table 4:** Experimental results on the lung cancer segmentation dataset (mean ± standard deviation across five-fold cross-validation).

Method	IoU	mIoU	HD ↓	DSC	mAcc	Params (M)	Latency (ms)
UNet++	0.688±0.034	0.701±0.032	8.86±0.83	0.741±0.030	0.728±0.035	9.2	12.5
DeepLabv3+	0.714±0.030	0.727±0.028	8.31±0.74	0.758±0.027	0.744±0.031	43.5	12.7
Mask2Former	0.739±0.041	0.751±0.038	7.44±1.02	0.777±0.035	0.762±0.042	61.0	25.3
SegFormer	0.746±0.036	0.759±0.033	7.26±0.86	0.783±0.031	0.767±0.036	64.0	14.3
SegNeXt	0.763±0.028	0.775±0.026	6.58±0.63	0.798±0.025	0.782±0.028	48.9	16.1
VWFormer	0.769±0.026	0.780±0.024	6.37±0.57	0.801±0.022	0.788±0.026	68.9	20.8
nnU-Net	0.772±0.022	0.784±0.020	6.21±0.48	0.804±0.019	0.789±0.022	31.2	18.6
SegMan	0.777±0.027	0.789±0.024	5.79±0.61	0.807±0.022	0.804±0.026	92.6	30.9
EDAFormer	0.779±0.023	0.790±0.021	5.88±0.49	0.809±0.020	0.799±0.023	65.8	19.4
Ours	0.788±0.018	0.799±0.017	5.28±0.36	0.816±0.016	0.811±0.018	67.2	17.4

As shown in Table 4, the proposed OncoSeg2D also achieved leading performance in various indicators on the Kaggle lung cancer segmentation dataset. In particular, it outperformed mainstream CNN and Transformer architectures in IoU, mIoU, and DSC, while maintaining the lowest value for the HD metric. This shows that this method not only has advantages in overall segmentation accuracy, but also exhibits stronger robustness and generalization capabilities in boundary modeling and morphology preservation, effectively addressing the complex changes of lung nodules and lesions in different clinical scenarios.

### 4.3 Ablation experiment results

This paper also presents ablation results, which an ablation analysis is performed on the baseline used in this paper. First, the module ablation results are presented, as shown in Table 5.

**Table 5:** Ablation study results on the Medical Segmentation Decathlon Challenge dataset and the lung cancer segmentation dataset (mean ± standard deviation across five-fold cross-validation).

Ablation settings	UBM	MPR	IoU	mIoU	HD ↓	DSC	mAcc
**Medical Segmentation Decathlon Challenge Dataset**							
Baseline (SegFormer)	–	–	0.830±0.034	0.843±0.031	5.61±0.74	0.881±0.030	0.855±0.034
Setting 1	✓	–	0.850±0.024	0.865±0.022	4.42±0.43	0.904±0.021	0.878±0.024
Setting 2	–	✓	0.858±0.021	0.873±0.019	3.98±0.36	0.912±0.018	0.890±0.021
Setting 3 (Ours)	✓	✓	**0.865±0.017**	**0.881±0.015**	**3.41±0.31**	**0.923±0.015**	**0.901±0.017**
**Lung cancer segmentation dataset**							
Baseline (SegFormer)	–	–	0.746±0.036	0.759±0.033	7.26±0.86	0.783±0.031	0.767±0.036
Setting 1	✓	–	0.775±0.024	0.787±0.022	5.92±0.47	0.804±0.021	0.797±0.024
Setting 2	–	✓	0.767±0.027	0.779±0.025	6.31±0.55	0.798±0.023	0.785±0.027
Setting 3 (Ours)	✓	✓	**0.788±0.018**	**0.799±0.017**	**5.28±0.36**	**0.816±0.016**	**0.811±0.018**

Ablation experiments show that introducing both the uncertainty-aware boundary modeling and morphology-preserving regularization modules significantly improves segmentation performance on two different datasets. For example, compared to the baseline model SegFormer, introducing either the uncertainty-aware boundary modeling or morphology-preserving regularization alone improves metrics such as IoU, mIoU, and DSC, while reducing HD, demonstrating the positive effects of both modules on feature extraction and boundary modeling. The performance gain of uncertainty-aware boundary modeling is more pronounced on boundary-sensitive metrics, reflecting its capability to refine ambiguous edge regions, whereas morphology-preserving regularization mainly enhances the global shape regularity and segmentation consistency.

Further examining the results for Setting 3, we find that this combination achieves optimal performance across all metrics: IoU, mIoU, and DSC reach the highest values, while HD is minimized, demonstrating strong complementarity between the two modules. This synergy indicates that uncertainty-aware boundary modeling focuses on local uncertainty refinement while morphology-preserving regularization constrains global morphology, resulting in a balanced improvement across precision and stability. Experiments on the lung cancer segmentation Dataset also confirm this conclusion. While the overall performance is lower than on the previous dataset, the combined uncertainty-aware boundary modeling and morphology-preserving regularization still significantly outperform both the individual modules and the baseline model. This fully demonstrates the robustness and effectiveness of the proposed method in different datasets and task scenarios.

### 4.4 Qualitative analysis of experimental results

In this section, we present the results of the segmentation experiment and the Grad-Cam experiment, also conducted on two datasets.

#### 4.4.1 Qualitative experimental results on the Medical Segmentation Decathlon Challenge dataset.

**Fig 6:**
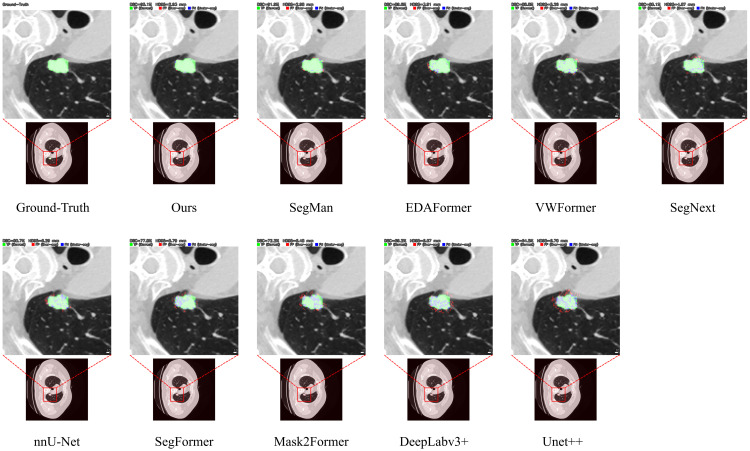
Medical Segmentation Decathlon Challenge dataset segmentation results.

First, we give the experimental results of the Medical Segmentation Decathlon Challenge dataset, as shown in Fig 6.

Experimental visualization results show that our proposed method demonstrates superior region coverage and boundary consistency across multiple case segmentation tasks. Compared to Mask2Former, Unet++, and Deeplabv3 + , our model achieves more complete coverage of lesion regions, avoiding the under-segmentation and over-segmentation observed in other methods. Furthermore, our method achieves closer alignment with the ground truth for boundary details, reducing jagged or irregular prediction errors, demonstrating its superior morphology preservation and edge refinement capabilities.

Further comparison of magnified regions reveals that while other models tend to miss or inaccurately segment small lesions or regions with blurred boundaries, our method more consistently captures this fine-grained information, ensuring smoother and more consistent segmentation results. This improvement not only enhances visual quality but also provides more reliable structural information for subsequent clinical analysis, demonstrating the practical value of our proposed model in real-world applications. Furthermore, this paper gives the Grad-Cam experimental results.

**Fig 7:**
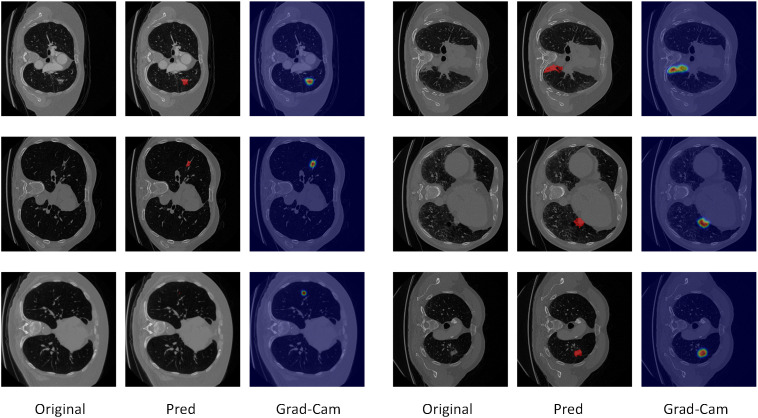
Medical Segmentation Decathlon Challenge dataset Grad-Cam experimental results.

Fig 7 show that the model achieves relatively accurate segmentation predictions on the Medical Segmentation Decathlon Challenge dataset, effectively covering lesion areas. Grad-Cam visualizations show distinct hotspots of interest, which are highly consistent with the distribution of the predicted mask. This demonstrates that the model not only generates reliable segmentation results at the output layer but also effectively focuses on lesion-related areas at the feature level, demonstrating strong interpretability and robustness, thereby enhancing its credibility for practical clinical applications.

#### 4.4.2 Qualitative experimental results on the lung cancer segmentation dataset.

**Fig 8:**
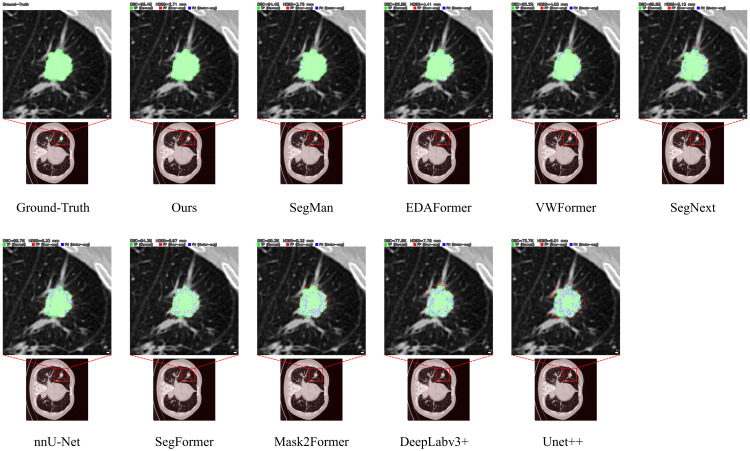
Segmentation results of the lung cancer segmentation dataset.

Similarly, this paper also gives the segmentation results of the lung cancer segmentation dataset and the Grad-Cam experimental results. First, the segmentation results are given, as shown in Fig 8.

The results in Fig 8 demonstrate that our method demonstrates strong robustness and accuracy in the task of lung cancer segmentation. A comparison with Ground-Truth shows that the model effectively captures the boundaries and morphology of lesion regions, maintaining high consistency, especially in the presence of blurred boundaries and low contrast. This demonstrates the model’s strong discriminative ability in lesion region identification and localization, mitigating overexpansion and omission issues.

Compared to other comparison methods, Mask2Former, Unet++, and Deeplabv3 + , our method demonstrates clearer lesion outlines and closer regional alignment. While Mask2Former and Unet++ perform reasonably well in gross segmentation, they are prone to incomplete edges and overprediction, while Deeplabv3 + ’s results tend to be more fuzzy. Overall, the proposed method strikes a balance between regional integrity and boundary accuracy, making it suitable for clinically assisted segmentation of lung CT nodules/tumors.

**Fig 9:**
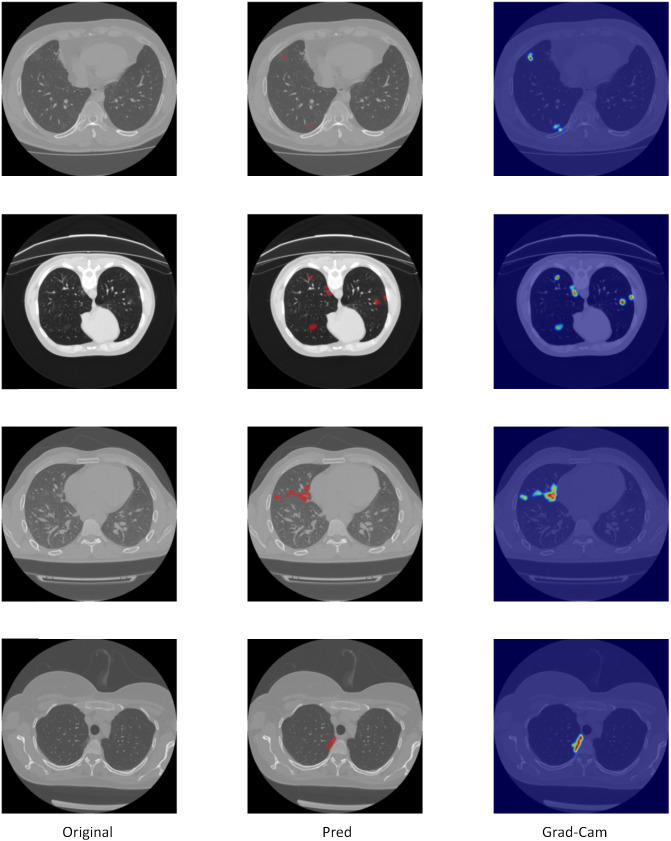
Lung cancer segmentation dataset Grad-Cam experimental results.

Finally, Grad-Cam is also used for analysis, and the experimental results are shown in Fig 9.

From the experimental results, it can be observed that Grad-Cam effectively highlights the critical regions of interest in lung CT images, particularly forming distinct high-intensity heatmap distributions around nodules or lesions, which are largely consistent with both the predicted results and the actual lesion areas. This indicates that the model demonstrates good interpretability in identifying lesion locations, with its decision-making focused on clinically relevant anatomical structures rather than irrelevant regions. At the same time, although certain samples still exhibit some blurring or incomplete coverage, the overall consistency between the Grad-Cam visualizations and the predictions provides strong support for the reliability of the model and its interpretability in clinical auxiliary diagnosis.

#### 4.4.3 Learning rate hyperparameter sensitivity experiment.

This paper also gives the experimental results of the learning rate hyperparameter sensitivity on two datasets, and the experimental results are shown in Table 6.

**Table 6:** Effect of learning rate on model performance across two datasets.

Dataset	Learning Rate	IoU	mIoU	HD ↓	DSC	mAcc
**Medical Segmentation Decathlon**	1e-3	0.816	0.829	6.63	0.868	0.849
	3e-4	0.846	0.860	4.87	0.897	0.876
	2e-4	0.859	0.873	3.96	0.913	0.891
	1e-4	**0.865**	**0.881**	**3.41**	**0.923**	**0.901**
**Lung cancer segmentation**	1e-3	0.719	0.731	8.47	0.752	0.739
	3e-4	0.754	0.766	6.88	0.788	0.774
	2e-4	0.773	0.784	5.96	0.802	0.794
	1e-4	**0.788**	**0.799**	**5.28**	**0.816**	**0.811**

The results presented in Table 6 demonstrate that the proposed OncoSeg2D framework maintains stable and consistent performance across different learning rates on both datasets. As the learning rate decreases from 1e-3 to 1e-4, the model shows a steady improvement in IoU, mIoU, and DSC, while the Hausdorff Distance continuously declines, indicating enhanced boundary precision and convergence stability. The optimal performance at 1e-4 learning rate on both the Medical Segmentation Decathlon and lung cancer segmentation datasets confirms the robustness of the proposed optimization strategy, suggesting that the model achieves a balanced trade-off between convergence speed and generalization ability under varying training conditions.

### 4.5 Restriction analysis

This paper also provides verification of the model’s generalization performance. Specifically, for two datasets, training is performed on one dataset and testing is performed on the other dataset. The experimental results are shown in Table 7.

**Table 7:** Cross-dataset transfer evaluation: lung cancer segmentation → Medical Segmentation Decathlon (top) and Medical Segmentation Decathlon → lung cancer segmentation (bottom).

Method	IoU	HD ↓	DSC
*Lung cancer segmentation → Medical Segmentation Decathlon*			
SegFormer	0.201	32.8	0.334
EDAFormer	0.228	30.7	0.371
SegMan	0.247	29.4	0.398
Ours	0.276	26.9	0.431
*Medical Segmentation Decathlon → lung cancer segmentation*			
SegFormer	0.238	34.6	0.384
SegMan	0.289	28.8	0.448
EDAFormer	0.294	29.1	0.453
Ours	0.329	24.1	0.496

From the cross-domain transfer results in Table 7, it is apparent that all models suffer a marked performance drop under out-of-domain evaluation, indicating that cross-dataset generalization in this task remains highly challenging. For example, in the Lung CT → MSD direction, SegFormer achieves an IoU of only 0.201, SegMan 0.247, and EDAFormer 0.228, while our model—although still performing the best comparatively—reaches only 0.276. Meanwhile, the HD values increase substantially, for example, SegFormer reaches an HD of 32.8, indicating a clear increase in boundary deviation and hard-to-segment outlier regions. The reverse transfer direction (MSD → Lung CT) exhibits a similar overall degradation, although the absolute metric values differ slightly. Notably, in both transfer directions, OncoSeg2D consistently maintains comparatively superior performance with lower HD values, suggesting that the proposed morphology-preserving regularization and uncertainty-aware modeling do contribute to robustness under cross-domain scenarios. Nevertheless, the absolute performance decline remains obvious, which also highlights an important limitation of the current framework for real-world deployment.

This limitation is not solely attributable to the model itself, but is mainly associated with the substantial domain gap between the two public datasets. Potential sources of this discrepancy include differences in imaging acquisition protocols, such as slice thickness, reconstruction kernels, and sampling intervals; variations in image intensity characteristics and preprocessing settings; differences in noise patterns caused by scanners, hospitals, and acquisition equipment; inconsistencies in annotation protocols and labeling criteria across datasets; and heterogeneity in lesion type, morphology, and size distribution. These factors jointly make it difficult for representations learned from the source domain to transfer effectively to the target domain. Therefore, the observed performance drop should be understood as a consequence of cross-dataset distribution mismatch, rather than as evidence of failure in the core model design.

## 5. Discussion

The proposed OncoSeg2D framework demonstrates strong potential for improving 2D CT lung cancer segmentation by jointly addressing two clinically persistent challenges, namely boundary ambiguity and morphology inconsistency. Compared with conventional CNN-based pipelines that primarily rely on local convolutional features, OncoSeg2D benefits from Transformer-style multi-scale contextual encoding, which helps stabilize predictions under complex appearance variations. More importantly, rather than treating lesion borders as deterministic targets, the uncertainty-aware boundary modeling explicitly characterizes ambiguous transition regions, which is particularly relevant for low-contrast tumors and indistinct interfaces that commonly lead to overconfident and jittery contours in existing methods. This design is conceptually related to the broader line of uncertainty-aware medical segmentation, including Bayesian and Monte-Carlo dropout-based uncertainty estimation, probabilistic segmentation networks that model predictive distributions, and evidence-based learning that quantifies confidence from model outputs. In contrast to approaches that primarily use uncertainty as a post-hoc diagnostic signal, our boundary modeling integrates uncertainty directly into boundary supervision to down-weight unreliable transition pixels and guide the network to focus on stable boundary evidence. In contrast to Transformer-based backbones such as SegFormer that emphasize efficient multi-scale representation but provide limited explicit boundary uncertainty handling, and edge-enhanced variants such as EDAFormer that strengthen edge responses in a largely deterministic manner, OncoSeg2D introduces a complementary mechanism that makes the model cautious where the evidence is weak while remaining decisive where boundary cues are clear. Meanwhile, morphology-preserving regularization injects global structural priors into the optimization process, mitigating fragmented regions, holes, and shape distortions that are often observed when purely pixel-wise objectives dominate training. From a clinical standpoint, the observed segmentation gains are most meaningful when they translate into more stable contour delineation across follow-up scans, improved agreement in lesion extent measurement, and reduced variability in downstream quantitative endpoints that rely on accurate boundaries and plausible shapes.

Nevertheless, it is important to clarify when OncoSeg2D may fail or show diminished benefit. Very small lesions or extremely thin structures may still be challenging because limited pixel support can amplify class imbalance and make both boundary evidence and geometric constraints less informative. Similarly, severe motion artifacts, beam hardening, or unusually aggressive reconstruction kernels can corrupt local gradients and intensity transitions, potentially reducing the effectiveness of boundary uncertainty modeling. In addition, strong domain shifts across institutions, scanners, and acquisition protocols may cause systematic intensity and texture changes that are not fully corrected by standard normalization, leading to reduced robustness on out-of-distribution cohorts. These cases highlight the practical need for careful quality control, domain harmonization, and broader multi-center validation before clinical deployment.

Despite these advantages, several limitations should be acknowledged. First, although we adopted patient-level data splitting to avoid leakage, the slice-wise modeling paradigm does not fully exploit inter-slice anatomical continuity, and performance computed at the slice level may underestimate uncertainty due to within-volume correlation; this motivates future extensions toward 3D or 2.5D modeling and patient-wise evaluation protocols that better reflect clinical usage. Second, domain shifts caused by heterogeneous scanners, reconstruction kernels, and acquisition protocols can still degrade robustness, suggesting the need for dedicated domain adaptation or harmonization strategies to further improve generalization in real-world deployment. Third, the computational cost–benefit trade-off should be considered for clinical workflows: while uncertainty-guided boundary supervision and projection-based geometric regularization improve contour reliability and shape plausibility, they introduce additional training-time overhead and modest inference-time complexity compared with a plain backbone. In practice, this added cost may be justified in scenarios where accurate delineation directly affects measurement consistency and treatment planning, whereas high-throughput screening settings may prefer lighter variants; thus, streamlined operator implementations and acceleration strategies (e.g., lightweight approximations or distillation) are promising directions. Finally, broader validation on multi-center cohorts and diversified lesion types would strengthen evidence for clinical translation, and integrating complementary modalities or clinical metadata may further enhance interpretability and decision support. Overall, OncoSeg2D offers a meaningful step toward bridging algorithmic performance and clinical applicability by improving both boundary reliability and structural plausibility, providing a promising direction for trustworthy and explainable medical image analysis.

## 6. Conclusion

This paper proposes OncoSeg2D, a deep framework for 2D CT lung cancer segmentation. It integrates the multi-scale feature extraction capabilities of the SegFormer backbone, uncertainty-aware boundary modeling, and morphology-preserving regularization. Without relying on additional annotations or 3D reconstruction, this approach effectively mitigates boundary blurring and morphological corruption, thereby improving segmentation accuracy while enhancing structural consistency and clinical interpretability. Experimental results demonstrate that OncoSeg2D achieves superior performance on two datasets, demonstrating the effectiveness and applicability of the proposed method.

Future research will focus on three key areas: first, further optimizing the efficient implementation of the uncertainty-aware boundary modeling and morphology-preserving regularization modules to reduce computational overhead and improve inference speed; second, extending OncoSeg2D to 3D CT and multimodal medical imaging to enhance cross-modality robustness and generalization; and third, integrating uncertainty quantification with clinical decision support systems to promote the application of segmentation results in real-world clinical diagnosis, lesion tracking, and treatment planning. Through these efforts, OncoSeg2D is expected to play a broader role in the field of intelligent medical image analysis.

## Supporting information

S1 File Appendix. (DOCX)
