# Supplementary material for: OncoSeg2D: A deep framework for semantic segmentation of lung cancer in 2D CT scans
Source: PLoS One. 2026 Jul 2;21(7):e0348719. doi: 10.1371/journal.pone.0348719 (PMC13327268; doi:10.1371/journal.pone.0348719)
Supplement: S1 File — (DOCX) [file pone.0348719.s001.docx]

[Appendix.](pone.0348719_Latex.xhtml#BibRef967252866)

Table 8. Sensitivity analysis of optimizer choice on two datasets (mean ± standard deviation across five-fold cross-validation).

| **Medical Segmentation Decathlon Challenge dataset** | | | | | |
| --- | --- | --- | --- | --- | --- |
| **Optimizer** | **IoU** | **mIoU** | **HD** ↓ | **DSC** | **mAcc** |
| AdaGrad | 0.836 ±0.024 | 0.851 ±0.022 | 4.26 ±0.36 | 0.898 ±0.021 | 0.874 ±0.024 |
| SGD | 0.845 ±0.022 | 0.861 ±0.020 | 3.97 ±0.33 | 0.906 ±0.019 | 0.882 ±0.022 |
| Adam | 0.857 ±0.019 | 0.873 ±0.017 | 3.64 ±0.29 | 0.915 ±0.017 | 0.892 ±0.019 |
| AdamW | 0.865 ±0.017 | 0.881 ±0.015 | 3.41 ±0.31 | 0.923 ±0.015 | 0.901 ±0.017 |
| **Lung cancer segmentation dataset** | | | | | |
| **Optimizer** | **IoU** | **mIoU** | **HD** ↓ | **DSC** | **mAcc** |
| AdaGrad | 0.748 ±0.026 | 0.760 ±0.024 | 6.46 ±0.55 | 0.784 ±0.023 | 0.772 ±0.026 |
| SGD | 0.761 ±0.023 | 0.773 ±0.021 | 6.08 ±0.49 | 0.795 ±0.021 | 0.783 ±0.023 |
| Adam | 0.779 ±0.020 | 0.790 ±0.019 | 5.66 ±0.42 | 0.808 ±0.018 | 0.799 ±0.020 |
| AdamW | 0.788 ±0.018 | 0.799 ±0.017 | 5.28 ±0.36 | 0.816 ±0.016 | 0.811 ±0.018 |

Table 9. Sensitivity analysis of batch size on two datasets (mean ± standard deviation across five-fold cross-validation).

| **Medical Segmentation Decathlon Challenge dataset** | | | | | |
| --- | --- | --- | --- | --- | --- |
| **Batch Size** | **IoU** | **mIoU** | **HD** ↓ | **DSC** | **mAcc** |
| 8 | 0.853 ±0.021 | 0.869 ±0.019 | 3.78 ±0.32 | 0.911 ±0.019 | 0.889 ±0.021 |
| 24 | 0.842 ±0.023 | 0.858 ±0.021 | 4.02 ±0.35 | 0.903 ±0.020 | 0.880 ±0.022 |
| 32 | 0.859 ±0.018 | 0.875 ±0.017 | 3.57 ±0.28 | 0.917 ±0.016 | 0.894 ±0.018 |
| 16 | 0.865 ±0.017 | 0.881 ±0.015 | 3.41 ±0.31 | 0.923 ±0.015 | 0.901 ±0.017 |
| **Lung cancer segmentation dataset** | | | | | |
| **Batch Size** | **IoU** | **mIoU** | **HD** ↓ | **DSC** | **mAcc** |
| 8 | 0.770 ±0.022 | 0.781 ±0.021 | 5.91 ±0.47 | 0.801 ±0.020 | 0.791 ±0.022 |
| 24 | 0.758 ±0.024 | 0.770 ±0.022 | 6.18 ±0.51 | 0.791 ±0.021 | 0.780 ±0.023 |
| 32 | 0.781 ±0.019 | 0.792 ±0.018 | 5.61 ±0.41 | 0.810 ±0.017 | 0.801 ±0.019 |
| 16 | 0.788 ±0.018 | 0.799 ±0.017 | 5.28 ±0.36 | 0.816 ±0.016 | 0.811 ±0.018 |

Fig 10. Comparison of pixel values in lesion areas.
